# Economic burden and patient-related determinants of medication waste in Saudi Arabia: evaluating re-dispensing initiatives for resource optimization

**DOI:** 10.3389/fphar.2026.1823795

**Published:** 2026-05-25

**Authors:** Mona Y. Alsheikh, Hana A. Althobaiti, Hussein M. Farag, Abdulaziz A. AlQahtani, Abdulaziz O. Baghdadi, Yazeed M. Binishaq, Moayad E. Albaqami, Sayraa A. Alshaikh

**Affiliations:** 1 Department of Pharmacy Practice, Faculty of Pharmacy, King Abdulaziz University, Jeddah, Saudi Arabia; 2 Department of Pharmaceutical Practices, College of Pharmacy, Umm Al-Qura University, Makkah, Saudi Arabia; 3 Pharmaceutical Sciences Department, Fakeeh College for Medical Sciences, Jeddah, Saudi Arabia; 4 Faculty of Pharmacy, King Abdulaziz University, Jeddah, Saudi Arabia

**Keywords:** economic burden, medication waste, over-prescription, pharmaceutical disposal, re-dispensing, Saudi Arabia, sustainability

## Abstract

**Introduction:**

Global medicine use rose 14% in 5 years, causing waste, financial loss, environmental damage, and limited access to therapies. Re-dispensing unused medications is possible, subject to legal and safety restrictions.

**Objective:**

To estimate the economic cost of medication waste, characterize patient-related determinants associated with waste, and assess the potential for re-dispensing based on the quality of the returned medications.

**Methods:**

A cross-sectional survey was conducted at King Abdulaziz University Hospital, Jeddah, Saudi Arabia, in December 2024. Unused medications were collected and categorized by use status, dosage form, Anatomical Therapeutic Chemical (ATC) system, and market value. Patient demographics and disposal practices were recorded via structured questionnaires.

**Results:**

A total of 206 patients returned 2,633 medications, which were primarily unused (64.98%) and in solid dosage forms (80.59%). Participants were predominantly female (59.22%) and aged 20–29 years (54.37%). Over-prescription was the leading cause of waste (70.87%). While 83.50% of participants possessed excess medications, 62.14% had never returned them previously, and 64.08% had disposed of them in the trash. The most frequent therapeutic classes were the alimentary tract and metabolism (31.41%), cardiovascular (14.93%), and nervous system (11.51%). Anti-neoplastic agents contributed the highest cost at 57,979.30 SAR (15,461.15 United States Dollars), despite representing only 0.38% of items. The total economic value of returned medications was 220,272.97 SAR (58,739.46 USD).

**Conclusion:**

Medication waste represents a substantial economic burden and carries significant potential implications for environmental sustainability. While the high quality of returned products reveals a clear opportunity for recovery through re-dispensing, successful implementation will depend on robust legislative support and further economic evaluation. Ultimately, a combination of strategic interventions, such as shorter first fills and structured return programs, is essential to effectively mitigate waste.

## 1 Introduction

Saudi Arabia faces mounting difficulties in disposing of unwanted medications, a situation that impacts both the well-being of its citizens and the economic health of the country. The rapid expansion of the healthcare system in Saudi Arabia has increased access to medications. Government provides comprehensive health services and insurance widely available across the country with substantial pharmaceutical coverage and increased medication use. A substantial quantity of prescribed medications remained unused, a consequence of physicians over-prescribing, overly large medication packaging, and shifts in treatment plans. These changes occurred because patients encountered side effects, felt the medication was ineffective, or discontinued treatment once they began to feel better, despite considerable government investment (Naseralallah et al., 2023; Kardas et al., 2024; Adeyemo et al., 2024). This leads to unnecessary financial costs, waste of healthcare resources, and potential risk to patient health and the environment.

A large number of unused medications are kept in households in Saudi Arabia, and some of it is returned to pharmacies, some is disposed of as household waste. These actions imply a lack of public understanding, with a part of population being unaware of how to dispose of medications properly and the broader consequences of pharmaceutical waste. Other studies have looked at unused medications, such as the types of medications that people return to pharmacies and why they do not use them (Wang et al., 2024; Wang et al., 2025; Blackburn et al., 2024). However, data on patients in Saudi Arabia are limited; the demographics of patients who discard medications are not well understood, nor are the behaviors that lead to medication waste.

It is important to understand these variables in order to develop effective ways to reduce medication waste, and these will need to be tailored to the local context to fit prescribing practices, patient behavior, and the healthcare system. Medication waste is a global problem, and as global use of medicines increases, it is estimated that by 2029, the number of daily doses of medicines will be approximately 3.7 trillion, even if the rate of increase is a low 4%. This reduced growth rate is associated with economic downturns in developed and developing economies (IQVIA Institute for Human Data Science, 2025). Pharmaceutical companies often sell products in prepackaged quantities that far exceed what is needed by patients, and physicians may write prescriptions for additional medication to continue treatment, even if a patient stops taking it early. The consequence of all this is a large quantity of unused, expired medications, placing a considerable economic burden on healthcare facilities (OECD, 2025).

In some nations, unopened and high-quality returned medications are looked at for re-dispensing as a way to minimize waste and preserve resources. Yet, the broad adoption of such initiatives has been restricted by safety worries, legal accountability concerns, inadequate quality assurance mechanisms, and regulatory obstacles (Smale et al., 2024; Watts et al., 2024; Wang et al., 2025). To date, the feasibility and acceptability of medication re-dispensing have not been well explored in Saudi Arabia despite the relevance of these initiatives to national healthcare sustainability objectives.

Conventional wastewater treatment often struggles to eliminate pharmaceutical residues, leading to their detection in low concentrations across water, soil, and living organisms globally. These persistent compounds can exert biologically active effects on both ecosystems and human health (Aziz et al., 2025; Bano et al., 2025). A prime illustration of this is the veterinary use of the nonsteroidal anti-inflammatory drug diclofenac, which has inflicted kidney toxicity upon Asian Gyps vultures, thus dramatically shrinking their numbers due to renal damage sustained from consuming carcasses of treated livestock (Swan et al., 2006). Despite regulatory bans, the presence of antibiotic residues in the environment exerts selective pressure on microbial communities, fostering the selection and spread of antimicrobial resistance, a significant and escalating global health concern (Murray et al., 2024).

This necessitates the use of sustainable approaches to handling medication waste, and thus, the main purpose of the current study was to determine the economic impact of medication waste in Saudi Arabia, identify the most significant contributing factors, and focus on patient-related demographic and behavioral determinants, promote appropriate medication disposal, and increase pzublic awareness of the economic and environmental costs of waste, as well as the quality of the returned medications to assess the feasibility of re-dispensing initiatives to support strategies that minimize waste, optimize medication use, and maximize the efficiency of healthcare resource allocation.

## 2 Materials and methods

### 2.1 Study design and setting

A questionnaire-based cross-sectional study was conducted at King Abdulaziz University Hospital (KAUH) in Jeddah, Saudi Arabia, from December 8 to 19 December 2024. The initiative aimed to raise awareness about the financial and potential environmental implications of medication waste and to encourage patients, their families, and visitors to donate and dispose of medications responsibly. As part of the campaign’s strategic deployment, a special booth was established in the hospital’s main lobby to actively engage participants. Patients and visitors passing through were invited to stop by, return any medications they no longer needed, and complete a brief questionnaire. Pharmacy interns, who had received specialized training for this project, effectively manned the booth to promote voluntary participation and assist with the completion of the concise questionnaire upon the return of medications.

### 2.2 Study procedure and participant recruitment

The study procedure was systematically divided into two distinct parts: participant information collection and medication donation processing.

#### 2.2.1 Participant information collection

A concise, pre-designed questionnaire was developed to capture relevant participant data aligned with the study’s objectives. Participants were recruited voluntarily during their hospital visits. Upon providing consent, individuals completed the survey, which elicited information regarding: 1) Basic demographics, including age, gender, and their relationship to the patient (patient or relative); 2) The primary reason for returning medications, categorized as overprescription and excess (quantities exceeding clinical need or surplus accumulation), prescription adjustments (clinical changes in therapy, dosage, or discontinuation), or death; 3) The duration for which medications were dispensed (more than 3 months, one to 3 months, less than 1 month, or not sure); 4) Previous history of incomplete medication use (yes or no); 5) Prior experience with returning unused medications (yes or no); and 6) Disposal practices, specifically regarding whether valid medications had been discarded in the trash (yes or no). All participants provided informed consent, and their confidentiality and anonymity were ensured. Data were collected anonymously in an Excel file from Google Forms.

#### 2.2.2 Medication donation processing

The second component of the study focused on the collection and classification of donated medications. Medications returned by participants were categorized into three types: 1) Unused medicines: unexpired medications that were prescribed but never initiated; 2) Partially used medicines: unexpired medications that were started but not fully consumed; and 3) Expired medicines: those that had passed their expiration date.

Upon receipt, well-trained pharmacy interns meticulously sorted and documented each returned medication. The classification was based on the following attributes: 1) Dosage form: including solid (tablets, capsules, powders, lozenges), liquid (solutions, syrups, suspensions, emulsion, elixirs), or semi-Solid (gels, creams, ointments); 2) Anatomical Therapeutic Chemical (ATC) classification system: The first level of this system was used; 3) Name: Both generic and brand names were recorded to facilitate the identification of duplicates; 4) Strength: referring to the labeled concentration of the active ingredient; 5) Supply status: Returned medications (both generic and brand) were cross-referenced against the National Unified Procurement Company (NUPCO) shortage list to check for any that were in short supply; 6) Number of retrieved units: The exact number of remaining items (e.g., tablets, capsules) in each package was recorded; 7) Number of units originally dispensed: recorded to determine the proportion of medication utilized.

Following classification, the medications followed specific pathways according to their condition and expiration status. To maximize social utility and minimize waste, all unused and unexpired medications were transferred to the Charitable Pharmacy at King Abdulaziz University Hospital for potential re-dispensing to eligible patients under institutional safety guidelines. Conversely, all expired medications were repurposed for educational and simulation-based training at the Faculty of Pharmacy. This structured approach ensured that every collected item was put to beneficial use rather than being discarded as environmental waste.

#### 2.2.3 Prices of returned medication

Prices for all returned medications were determined based on their current retail market value. The following cost definitions and calculations were applied: 1) Price per medication box: This was defined as the original retail price of a complete, unopened medication box, based on the number of units it contained; 2) Price per medication unit: The cost of a single unit (e.g., one tablet or capsule) was calculated by dividing the medication box price by the total number of original units in the box; 3) Total price of returned medications: This represents the total economic value of the medication waste. It was calculated by multiplying the price per unit by the actual number of units retrieved during the campaign.

### 2.3 Data and cost analyses

Statistical analyses were conducted using Microsoft Excel (Microsoft Corporation, Redmond, WA, United States; version 16.0), IBM® SPSS Statistics version 28.0 (IBM Corp., Armonk, NY, United States), and Rstudio statistical software (version 4.3.1). Descriptive statistics, presented as frequencies (n), percentages (%), mean (M), standard deviation (SD), median (Mdn), and interquartile range (IQR), were utilized to summarize the participants’ demographics, reasons for returning medications, disposal practices, and the classification and economic value of the collected medications. The monetary value of all returned medications was systematically calculated based on their retail market prices, reported in both Saudi Riyals (SAR) and United States Dollars (USD). A generalized linear model (GLM) utilizing a gamma distribution and log link function was utilized to assess the economic burden of medication waste. The main outcome was the total waste cost per medication record, analyzed against independent variables including medication status (used, unused, or expired), therapeutic class (ATC system), dosage form, market availability status (brand and/or generic availability), and the number of units retrieved. All regression analyses employed two-tailed tests, with statistical significance set at *p* < 0.05.

## 3 Results

A total of 206 participants participated during the campaign. The majority of participants were female (122, 59.22%), with their average age being M = 33.77 years, SD ± 14.30. The largest age group was 20–29 years, comprising over half of the participants (112, 54.37%). In terms of their relationship to the patient, a significant majority were relatives returning medications (140, 67.96%). The primary reason for returning medications was overwhelmingly due to over-prescription and excess supply (146, 70.87%), followed by prescription adjustments (44, 21.36%) and patient death (16, 7.77%). Furthermore, while most participants (172, 83.50%) reported having leftover medications, a considerable portion (128, 62.14%) had never previously returned medications to a pharmacy. The majority of participants (132, 64.08%) reported previously disposing of unused, valid medications in the trash (Table 1). A total of 2,633 returned medications were categorized based on their status, with the majority being unused (1,711; 64.98%), expired (571; 21.69%), and partially used (351; 13.33%) (Table 2). Furthermore, the majority of returned medications were solid dosage forms (2,122; 80.59%), followed by liquid dosage forms (355; 13.48%). Semi-solids (gels and creams) made up a small portion of the total (156; 5.92%) (Table 3).

**TABLE 1:** Participant demographic and behavioral characteristics (*N* = 206).

Characteristic	Frequencies (n)	Percentages (%)
Gender		
Male	84	40.78%
Female	122	59.22%
Age (Years)		
Mean Age	33.77	
Standard Deviation	±14.30	
Age group distribution		
20–29 years	112	54.37%
30–39 years	24	11.65%
40–49 years	36	17.48%
50–59 years	14	6.80%
60–69 years	16	7.77%
70+ years	4	1.94%
Relationship to the patient		
Patient	66	32.04%
Relative	140	67.96%
Primary reason for returning medications		
Overprescription and Excess	146	70.87%
Prescription Adjustments	44	21.36%
Death	16	7.77%
Duration for which medications were dispensed		
More than 3 months	42	20.39%
One to 3 months	62	30.10%
Less than 1 month	34	16.50%
Not Sure	68	33.01%
Have you ever had medications that you did not use completely?		
Yes	172	83.50%
No	34	16.50%
Have you ever returned unused medications?		
Yes	78	37.86%
No	128	62.14%
Have you ever disposed of valid medications by throwing them in the trash?		
Yes	132	64.08%
No	74	35.92%

N represents the total number of participants; data are presented as frequency (n) and percentage (%). M ± SD, represents the Mean ± Standard Deviation for age.

**TABLE 2:** Classification of returned medications by medication status (*N* = 2,633).

Dosage forms	Frequencies (n)	Percentages (%)
Unused	1,711	64.98%
Expired	571	21.69%
Partially used	351	13.33%

N represents the total number of participants; data are presented as frequency (n) and percentage (%).

**TABLE 3:** Classification of returned medications by dosage forms (*N* = 2,633).

Dosage forms	Frequencies (n)	Percentages (%)
Liquid (solutions, syrups, suspensions, emulsions, elixirs)	355	13.48%
Semi-solid (gels, creams, ointments)	156	5.92%
Solid (tablets, capsules, powders, lozenges)	2,122	80.59%

N represents the total number of participants; data are presented as frequency (n) and percentage (%).

The returned medications were also classified according to the ATC system. The majority of the alimentary tract and metabolism (A) class (827; 31.41%), the cardiovascular system (C) class (393; 14.93%), and the nervous system (N) class (303; 11.51%), accounting for over 50% of the total. Anti-neoplastic and immunomodulating agents (L) (10; 0.38%) and antiparasitic products (P) (3; 0.11%) were among the least returned classes (Table 4).

**TABLE 4:** Classification of returned medications by ATC system (*N* = 2,633).

ATC class name	Frequencies (n)	Percentages (%)
A: Alimentary tract and metabolism	827	31.41%
B: Blood and blood forming organs	108	4.10%
C: Cardiovascular system	393	14.93%
D: Dermatologicals	136	5.17%
G: Genito urinary system and sex hormones	89	3.38%
H: Systemic hormonal preparations, excluding sex hormones and insulins	40	1.52%
J: Anti-infectives for systemic use	129	4.90%
L: Anti-neoplastic and immunomodulating agents	10	0.38%
M: Musculo-skeletal system	231	8.77%
N: Nervous system	303	11.51%
P: Antiparasitic products, insecticides, and repellents	3	0.11%
R: Respiratory system	238	9.04%
S: Sensory organs	71	2.70%
V: Various	55	2.09%

N represents the total number of participants; data are presented as frequency (n) and percentage (%). ATC: anatomical therapeutic chemical.

The most frequently returned medications by generic name were metformin (159; 6.04%), followed by rivastigmine (153; 5.81%), and atorvastatin (83; 3.15%). Furthermore, a significant portion of the returned items consisted of combination products (Table 5).

**TABLE 5:** Most frequently returned medications by generic name (*N* = 2,633).

Generic name	Frequencies (n)	Percentages (%)
Metformin	159	6.04%
Rivastigmine	153	5.81%
Atorvastatin	83	3.15%
Empagliflozin	68	2.58%
Rosuvastatin	48	1.82%
Pantoprazole	46	1.75%
Paracetamol	43	1.63%
Furosemide	40	1.52%
Montelukast sodium	40	1.52%
Esomeprazole	39	1.48%
Allopurinol	38	1.44%
Diclofenac	38	1.44%
Bisoprolol fumarate	37	1.41%
Mebeverine	35	1.33%
Celecoxib	34	1.29%
Domperidone	32	1.22%
Vitamin B1/B6/B12	32	1.22%
Omeprazole	31	1.18%
Amoxicillin/Clavulanic acid	30	1.14%
Amlodipine	29	1.10%
Paracetamol/Chlorzoxazone	16	0.61%
Metformin/Empagliflazon	14	0.53%
Paracetamol/Codeine phosphate/Caffeine	12	0.46%
Atorvastatin/Ezetimibe	8	0.30%
Amoxicillin	3	0.11%
Amlodipine/Valsartan	3	0.11%
Omeprazole/Sodium bicarbonate	2	0.08%
Metformin/Sitagliptin	1	0.04%
Paracetamol/Orphenadrine citrate	1	0.04%
Paracetamol/Pseudoephedrine/Diphenhydramine	1	0.04%
Paracetamol/Caffeine	1	0.04%

N represents the total number of participants; data are presented as frequency (n) and percentage (%).

The most frequently returned medications by brand name were Rivetal (151; 5.73%), followed by Jardiance (68; 2.58%), Glucare XR (60; 2.28%), and Glucare (52; 1.97%). Other commonly returned brands included No-Uric, Atorlip, and Nexium, each accounting for more than 1% of the total (Table 6).

**TABLE 6:** Most frequently returned medications by brand name (*N* = 2,633).

Brand name	Frequencies (n)	Percentages (%)
Rivetal	151	5.73%
Jardiance	68	2.58%
Glucare XR	60	2.28%
Glucare	52	1.97%
No-uric	38	1.44%
Atorlip	28	1.06%
Nexium	28	1.06%
Xatral XL	27	1.03%
Dompy	26	0.99%
Ivarin	24	0.91%
Airfast	23	0.87%
Diusemide	23	0.87%
Concor	22	0.84%
Tobrazole	22	0.84%
Omipure	21	0.80%
Glucophage	19	0.72%
Movicol	19	0.72%
Dermovate	18	0.68%
Neuro-B	18	0.68%
Adol	17	0.65%
Metfor	17	0.65%
Predo	17	0.65%

N represents the total number of participants; data are presented as frequency (n) and percentage (%).

The total economic value of all returned medications was estimated at 220,272.97 SAR (58,739.47 USD). The cost distribution was highly skewed, with most of the value coming from a limited number of items. The total value is high, but the median total price per medication was 36.45 SAR (9.72 USD), with an IQR = of 83.05 SAR (22.15 USD). This indicates that most returned medications were relatively inexpensive, high-volume products. The high total cost, on the other hand, is caused by expensive “outlier” medications, such as specialty oral oncolytics and immunomodulators. In contrast, the high total cost is driven by high-priced “outlier” medications, specifically specialty oral oncolytics and immunomodulators (Table 7).

**TABLE 7:** Descriptive statistics of returned medication prices.

Measure	Total price (box)	Total price (Unit)	Total price
Total	140,954.2 SAR	24,367.03 SAR	220,272.97 SAR
	37,587.79 USD	6,497.87 USD	58,739.47 USD
Mdn	34.55 SAR	2.16 SAR	36.45 SAR
	9.21 USD	0.58 USD	9.72 USD
IQR	70.17 SAR	3.75 SAR	83.05 SAR
	18.71 USD	1.00 USD	22.15 USD

Mdn: Median; IQR: interquartile range; SAR: saudi riyals; USD: united states dollars.

The total value of unused medications was the highest, at SAR 179,032.49 (USD 47,742.00), accounting for the largest share of the total medication waste cost. Expired medications contributed SAR 28,733.06 (USD 7,662.15), while partially used medications were valued at SAR 12,507.42 (USD 3,335.31). The total cost of returned medication was predominantly driven by unused medications that the patient never used (Table 8).

**TABLE 8:** Descriptive statistics of returned medication prices by medication status.

Medication status	Total price (box)	Total price (Unit)	Total price
Expired	17,778.73 SAR	3,403.20 SAR	28,733.06 SAR
	4,740.99 USD	907.52 USD	7,662.15 USD
Unused	105,079.88 SAR	19,347.11 SAR	179,032.49 SAR
	28,021.3 USD	5,159.23 USD	47,742.00 USD
Partially used	18,095.57 SAR	1,616.71 SAR	12,507.42 SAR
	4,825.48 USD	431.12 USD	3,335.31 USD

SAR: saudi riyals; USD: united states dollars.

The highest total cost was associated with the anti-neoplastic and immunomodulating agents (L) class, which accounted for SAR 57,979.30 (USD 15,461.14), despite representing only 10 items (0.38%). This was followed by the alimentary tract and metabolism (A) class at SAR 47,910.08 (USD 12,776.02) and the nervous system (N) class at SAR 23,436.71 (USD 6,249.78). In contrast, classes like antiparasitic products (P) and systemic hormonal preparations (H) had the lowest total costs, indicating a lower economic burden from waste in these areas. This analysis shows that a small number of high-cost medications from specific therapeutic classes account for the greatest share of the total monetary value of medication waste (Table 9).

**TABLE 9:** Descriptive statistics of returned medication prices by ATC system.

ATC class name	Total price (box)	Total price (Unit)	Total price
A: Alimentary tract and metabolism	34,746.61 SAR	8,221.08 SAR	47,910.08 SAR
	9,265.76 USD	2,192.28 USD	12,776.02 USD
B: Blood and blood forming organs	6,038.59 SAR	1,415.34 SAR	13,449.09 SAR
	1,610.29 USD	377.42 USD	3,586.42 USD
C: Cardiovascular system	15,365.25 SAR	519.63 SAR	21,093.75 SAR
	4,097.4 USD	138.56 USD	5,625.00 USD
D: Dermatologicals	2,935.05 SAR	2,628 0.43 SAR	3,877.20 SAR
	782.68 USD	700.91 USD	1,033.92 USD
G: Genito urinary system and sex hormones	6,208.54 SAR	217.27 SAR	7,654.27 SAR
	1,655.61 USD	57.93 USD	2,041.14 USD
H: Systemic hormonal preparations, excluding sex hormones and insulins	6,525.5 SAR	2,420.51 SAR	9,728.65 SAR
	1,740.13 USD	645.47 USD	2,594.30 USD
J: Anti-infectives for systemic use	7,132.6 SAR	1,059.21 SAR	7,705.51 SAR
	1,902.02 USD	282.45 USD	2,054.80 USD
L: Anti-neoplastic and immunomodulating agents	20,483.95 SAR	339.04 SAR	57,979.3 SAR
	5,462.38 USD	90.41 USD	15,461.14 USD
M: Musculo-skeletal system	8,595.64 SAR	2,364.56 SAR	12,109.52SAR
	2,292.17 USD	630.54 USD	3,229.20 USD
N: Nervous system	20,679.57 SAR	947.04 SAR	23,436.71 SAR
	5,514.55 USD	252.54 USD	6,249.78 USD
P: Antiparasitic products, insecticides, and repellents	180.3 SAR	3.38 SAR	131.23 SAR
	48.08 USD	0.90 USD	34.99 USD
R: Respiratory system	8,390.60 SAR	3,048.46 SAR	10,094.06 SAR
	2,237.49 USD	812.92 USD	2,691.75 USD
S: Sensory organs	1,204.3 SAR	791.97 SAR	2,463.68 SAR
	321.14 USD	211.19 USD	656.98 USD
V: Various	2,467.66 SAR	391.05 SAR	2,639.87 SAR
	658.04 USD	104.28 USD	703.96 USD

ATC: anatomical therapeutic chemical; SAR: saudi riyals; USD: united states dollars.

The most expensive returned medication was abiraterone acetate (Zytiga), a cancer medication, which had a total price of SAR 40,117.80 (USD 10,698.08) for the returned quantity. Other high-value returns included dimethyl fumarate (Sclera), a multiple sclerosis medication, valued at SAR 11,110.50 (USD 2,962.80), and Darbepoetin alfa (Aranesp), which had a total return value of SAR 7,754.70 (USD 2,067.92) across six boxes. The high costs associated with these few returned medications underscore the considerable economic burden of medication waste, particularly for high-cost specialized treatments. The data also reveal that most of these expensive medications were returned unused, highlighting a major opportunity for cost savings through re-dispensing programs (Table 10).

**TABLE 10:** The most expensive medications returned by participants.

Dosage form	Medication count (boxes)	Generic name	Brand name	Strength	ATC	Medication status	Original number of units/boxes	Retrieved number of units	Total price (box)	Total price (Unit)	Total price
Solid	4	Abiraterone acetate	Zytiga	250 mg	L	Unused	120	480	10,029.5 SAR	83.58 SAR	40,117.80 SAR
									2,674.52 USD	22.29 USD	10,698.08 USD
Solid	1	Fingolimod	Fegona	0.5 mg	L	Unused	28	28	4,806.25 SAR	171.65 SAR	4,806.25 SAR
									1,281.67 USD	45.77 USD	1,281.67 USD
Solid	1	Valganciclovir	Valgan	450 mg	J	Partially used	60	44	2,499.85 SAR	41.66 SAR	1,833.22 SAR
									666.63 USD	11.11 USD	488.86 USD
Solid	3	Dimethyl fumarate	Sclera	240 mg	L	Unused	56	168	3,703.50 SAR	66.13 SAR	11,110.50 SAR
									987.60 USD	17.64 USD	2,962.80 USD
Liquid	1	Darbepoetin alfa	Aranesp	60 µg	B	Unused	4	4	1,909.35 SAR	477.34 SAR	1,909.35 SAR
									509.16 USD	127.29 USD	509.16 USD
Liquid	3	Romosozumab	Evenity	105 Mg/1.17 mL	M	Unused	2	6	1,808.99 SAR	904.50 SAR	5,426.97 SAR
									482.40 USD	241.20 USD	1,447.19 USD
Liquid	1	Teriparatide	Forteo	20 µg/80 µL	H	Unused	1	1	1,421.95 SAR	1,421.95 SAR	1,421.95 SAR
									379.19 USD	379.19 USD	379.19 USD
Liquid	6	Darbepoetin alfa	Aranesp	40 µg	B	Unused	4	24	1,292.45 SAR	323.11 SAR	7,754.70 SAR
									344.65 USD	86.16 USD	2,067.92 USD
Solid	1	Capecitabine	Capecitabine SPC	500 mg	L	Unused	120	120	1,060.55 SAR	8.84 SAR	1,060.55 SAR
									282.81 USD	2.36 USD	282.81 USD
Liquid	1	Denosumab	Prolia	60 mg/mL	M	Unused	1	1	974.00 SAR	974.00 SAR	974.00 SAR
									259.73 USD	259.73 USD	259.73 USD
Solid	1	Tacrolimus	Prograf	1 mg	L	Unused	100	100	884.20 SAR	8.84 SAR	884.20 SAR
									235.79 USD	2.36 USD	235.79 USD
Solid	15	Semaglutide	Ozempic	1 mg	A	Unused	1	15	418.65 SAR	418.65 SAR	6,279.75 SAR
									111.64 USD	111.64 USD	1,674.60 USD
Solid	1	Semaglutide	Ozempic	0.5 mg	A	Unused	1	1	418.65 SAR	418.65 SAR	418.65 SAR
									111.64 USD	111.64 USD	111.64 USD
Total price									31,227.64 SAR	5,318.90 SAR	83,997.69 SAR
									8,327.42 USD	1,418.37 USD	22,399.44 USD

ATC, anatomical therapeutic chemical; SAR, saudi riyals; USD, united states dollars.

The results from the multivariable Gamma generalized linear model analyzing medication-related determinants of medication waste cost showed that unused medications were linked to nearly twice the waste cost compared with used medications (cost ratio [CR] = 1.98; 95% CI: 1.56–2.49; p < 0.001), while expired medications resulted in a 55% higher waste cost (CR = 1.55; 95% CI: 1.16–2.07; p = 0.002). Substantial variation in waste cost was observed among therapeutic classes. Using ATC class A as the baseline, ATC class L showed considerably higher waste costs, with an estimated more than 30-fold increase (CR = 33.55; 95% CI: 9.00–309.81; p < 0.001). In contrast, ATC classes M and R were linked to notably lower waste costs, with reductions of around 31% (CR = 0.69; 95% CI: 0.51–0.94; p = 0.013) and 39% (CR = 0.61; 95% CI: 0.44–0.84; p = 0.002), respectively. There was no significant difference in waste costs for ATC class C compared with the reference. The dosage form was also a key factor affecting waste costs. Compared with liquid forms, semi-solid and solid forms had significantly lower waste costs, reductions of 70% (CR = 0.30; 95% CI: 0.19–0.48; p < 0.001) and 58% (CR = 0.42; 95% CI: 0.31–0.56; p < 0.001), respectively. Market availability status was strongly associated with economic waste. Relative to medications where both brand and generic products were available, a shortage of the generic version only was associated with more than a four-fold increase in waste costs (CR = 4.59; 95% CI: 2.08–9.48; p < 0.001). Furthermore, scenarios where neither version was in shortage nearly tripled the waste cost (CR = 2.81; 95% CI: 1.39–5.01; p = 0.001). No statistically significant difference in waste costs was observed in shortages affecting only the brand-name product. Also, the number of units retrieved was positively correlated with waste cost; each additional unit was associated with about a 1% increase in total waste cost (CR = 1.01; 95% CI: 1.01–1.01; p < 0.001), indicating a volume-dependent relationship (Table 11).

**TABLE 11:** Medication-related determinants of the economic burden of medication waste.

Predictor	*Cost ratio (CR)	95% confidence interval	*p* value
Intercept	23.77	12.37–50.51	<0.001
Medication status (ref: Used)			
Unused	1.98	1.56–2.49	<0.001
Expired	1.55	1.16–2.07	0.002
ATC therapeutic class (ref: ATC: A)			
C	0.95	0.72–1.26	0.714
L	33.55	9.00–309.81	<0.001
M	0.69	0.51–0.94	0.013
N	1.19	0.90–1.58	0.226
R	0.61	0.44–0.84	0.002
**Other	1.29	1.00–1.68	0.056
Dosage form (ref: Liquid)			
Semi-solid	0.30	0.19–0.48	<0.001
Solid	0.42	0.31–0.56	<0.001
Market availability status (ref: Both brand and generic available)			
Brand only (brand is in shortage)	1.54	0.73–2.97	0.217
Generic only (generic is in shortage)	4.59	2.08–9.48	<0.001
Neither (neither brand nor generic in shortage)	2.81	1.39–5.01	0.001
Retrieved number of units	1.01	1.01–1.01	<0.001

* Regression results are presented as cost ratios obtained by exponentiating model coefficients. Cost ratios greater than 1 indicate higher waste cost, while cost ratios less than 1 indicate lower waste cost relative to the reference category.

** Therapeutic classes with low frequency were combined into an “Other” category, which included ATC, groups B, D, G, H, J, P, S, and V.

## 4 Discussion

This study represents a pioneering, in-depth evaluation of medication waste in Saudi Arabia. It uniquely combines an examination of patient-linked factors, the financial impact, and the practicality of re-dispensing programs, all aligned with the nation’s sustainability objectives. The findings highlight that medication waste stems from a complex relationship of prescribing habits, patient actions, and the absence of structured systems for managing medications after they’ve been dispensed. This study aligns with prior studies from Saudi Arabia and other countries, indicating that women and younger individuals are primarily responsible for managing household medicines (Alqurshi, 2020; Jafarzadeh et al., 2021). Remarkably, over half of the respondents had returned medicines prescribed to other family members, consistent with household storage practices and informal caregiving dynamics that could increase the risk of inappropriate use, waste, and unsafe disposal.

Although reported adherence to medication was relatively high (83.5%), a substantial proportion of the respondents (64.08%) discarded usable medicines in household trash, and the rate of returning unused medications was moderate (62.14%), which shows that there are still gaps in patient education and disposal infrastructure (Alqurshi, 2020). Unlike earlier Saudi research, which pointed to insufficient knowledge as the primary driver of improper disposal (Alqurshi, 2020), this finding diverges. Globally, similar patterns emerge, as the crucial step of discarding medications is often overlooked, leading to significant repercussions for both the environment and public health (Jafarzadeh et al., 2021; World Health Organization (WHO), 2025). The waste was primarily caused by overstocking at the start and patients stopping their treatment. Nearly 60% reported having a one-to three-month supply, and 20% reported having more than 3 months, although many were unsure of their quantities. These results show some success in patient–provider communication, but also illustrate the ongoing disconnect between the quantities prescribed and the therapeutic need, especially for chronic conditions.

Under Vision 2030, Saudi Arabia has achieved considerable success in healthcare waste regulation, with the establishment of the National Center for Waste Management (NCWM), yet the current regulations address institutional healthcare waste, rather than household pharmaceuticals (National Center for Waste Management (NCWM), 2023). Public guidance on how to dispose of medication is scattered and mostly indolent, with people often having to search online instead of participating in hands-on, community-led initiatives (National Center for Waste Management (NCWM), 2023). The lack of a national medication take-back system reflects similar challenges around the world, and is an opportunity lost given the centralized healthcare governance of Saudi Arabia. In addition to environmental contamination, improper disposal of medications also leads to AMR, especially through the disposal of antibiotics, which the WHO and the U.S. EPA have identified as a global public health threat (U.S. Environmental Protection Agency (EPA), 2025; World Health Organization (WHO), 2025).

Most of the medications returned in this study (64.98%) were unconsumed, with fewer being expired (21.69%) or partially used (13.33%), pointing to over-ordering and premature cessation of use as the main drivers of waste, rather than problems with how they were kept or when they were disposed of. Notably, more than 80% of the returned items were solid oral dosage forms, which are the most stable pharmaceutical formulations and the best suited for quality-assured re-dispensing. The majority of returned units were low-cost medications, including amoxicillin/clavulanic acid and metronidazole, which are individually inexpensive but are used so widely that their environmental and public health risks, especially regarding AMR, are significant. Total waste costs were also driven by a few high-cost medicines that were returned infrequently, a pattern seen internationally where specialty therapies drive waste expenditures even though they are returned infrequently (Bekker et al., 2019; Smale et al., 2024). The returned medications were most concentrated in ATC classes A (Alimentary Tract and Metabolism), C (Cardiovascular), and N (Nervous System), and the most frequently returned medications—metformin, rivastigmine, atorvastatin, empagliflozin, and pantoprazole—reflect national prescribing patterns and epidemiological data, suggesting that waste occurs mainly in high-volume chronic therapies (Alhomoud, 2020). The return rate of less than 1% was lower than other medication groups, but the medications accounted for more than one-third of total waste costs, which is consistent with the global evidence that oncology and specialty therapies account for the largest economic losses from medication waste (World Health Organization (WHO), 2023). The lack of high-return medications like abiraterone acetate and dimethyl fumarate points to chances for focused interventions, particularly at the start of treatment when patients are most likely to stop.

Global insights confirm the viability of minimizing waste from quality-assured, unused medications through re-dispensing, especially for sealed, room-temperature oral solids, all while maintaining patient safety. A prime example is the Dutch Re-dispensing of Oral Anticancer Drugs (ROAD) program, which, once operational costs were factored in, resulted in a 68% decrease in waste and produced annual savings of €576–€1,591 for each patient (Bekker et al., 2019; Smale et al., 2024). Implementing re-dispensing programs in Saudi Arabia requires solid legal structures, pharmacist accreditation, comprehensive traceability, and public assurance. However, these initiatives perfectly match the sustainability targets of Vision 2030 and the WHO’s advice on managing pharmaceuticals sustainably (National Center for Waste Management, 2023; World Health Organization (WHO), 2025). By initiating pilot programs in major urban tertiary hospitals, focusing on eligible high-cost oral medications, the necessary data for feasibility and safety can be obtained before a nationwide expansion.

Overall, the findings suggest a dual approach for policy: implement upfront strategies to minimize waste, such as prescribing smaller initial quantities, performing early clinical assessments, and establishing protocols for discontinuing proton pump inhibitors and polypharmacy. Simultaneously, create robust take-back and redistribution systems to reclaim value from unavoidable waste (Saha et al., 2025; Wang et al., 2024). Despite the specific local factors shaping medication waste in this study, its fundamental causes mirror global trends: chronic disease drugs make up the largest portion of waste by volume, expensive specialty medications significantly contribute to costs, and disposal practices continue to be environmentally poor across the globe. Therefore, the Saudi approach offers valuable insights for other nations striving to harmonize patient safety with cost control and ecological responsibility.

### 4.1 Strengths and limitations

The study has several important strengths that increase its value and efficacy: it conducts an in-depth economic analysis of waste due to unused, expired, and partially used medications in Saudi Arabia, measures the quantity and cost of unused, expired, and partially used medications, identifies major contributing factors to waste through analysis of each medication category and high-value products, includes ATC classification and brand-level data for more detailed policy recommendations, places its findings within national priorities in health such as Saudi Vision 2030 and NCWM guidelines and international best practice such as ROAD in the Netherlands to make locally relevant and globally informed recommendations, and uses economic tactic of re-dispensing based on empirical data for easy adoption by healthcare planners and policymakers (Khan et al., 2025; World Health Organization (WHO), 2023; Smale et al., 2024).

Several limitations in this study must be considered: (1) the cross-sectional design limits the ability to establish causality between the factors identified and wastage medication, (2) the use of self-reported data introduces recall and social desirability bias, as the participants may not accurately report their activities, (3) the sample may not be representative of the entire Saudi population, especially rural or poor areas, and findings may not be generalizable, (4) it lacks clinical background data, such as diagnoses of patients or reasons for stopping medication, which could provide further information on etiology, (5) although environmental concerns are raised, no overt environmental analysis, such as measurements of pharmaceutical residues in water or soil, is included, (6) although the potential benefits of re-dispensing are discussed, no systematic cost-benefit analysis or feasibility study tailored to the Saudi health system is presented, (7) the study is limited to solid dosage forms of oral medications, omitting cold-chain and injectable medications, which also generate waste but present different challenges for re-dispensing, and (8) no views from other key stakeholders, including healthcare professionals, pharmacists, or policymakers, who need to be involved in designing and implementing effective measures to reduce wastage, are presented.

### 4.2 Prospects and recommendations

Potential research areas that could be explored to prevent medication waste include: piloting should determine if the re-dispensing programs are feasible (especially for high-cost room-temperature oral medications with longer shelf life), longitudinal studies are needed to demonstrate long-term effects of front-end interventions such as reduced first-fill amounts and deprescribing, qualitative patient caregiver pharmacist and prescriber can delineate drivers for wasteful behavior which could inform targeted educational campaigns; potential environmental implications assessments should quantify ecological impact from improper disposal especially antibiotics and biologics. Lastly, national policy frameworks may overcome the legal, logistical, and regulatory challenges of re-dispensing, including quality control, traceability, public awareness to reduce medication waste, optimizing healthcare efficiency, and promoting environmental sustainability, as part of Vision 2030 international best practices.

## 5 Conclusion

This study investigates the financial impact and determinants of inefficient medication utilization, alongside the potential for re-dispensing within Saudi Arabia. The findings indicate that a substantial portion of high-cost medications, specifically those used in oncology and chronic care, significantly contribute to overall wastage costs, which align with existing global research and necessitate focused interventions. The study highlighted that oversupply, discontinuation of therapy, and the absence of formal return channels were the main drivers of waste. The results showed that front-end interventions (such as shortened first fills, medication synchronization, and deprescribing recommendations) and back-end approaches (such as quality-tested re-dispensing) have the potential to reduce waste, and that a national take-back system, legal mandates, and public education campaigns are needed to ensure long-term management of medication waste. Although the study provides helpful information and policy recommendations, it is limited by its cross-sectional design and reliance on self-report data; future studies should be based on longitudinal assessments, stakeholder perceptions, and potential environmental implications analyses. This study adds to the evidence for systemic changes to prevent medicine waste, optimize healthcare efficiency, and align with Saudi Vision 2030 and global sustainability initiatives.

## Data Availability

The raw data supporting the conclusions of this article will be made available by the authors, without undue reservation.

## References

[B1] Adeyemo K. S. Mbata A. O. Balogun O. D. (2024). Pharmaceutical waste management and reverse logistics in the US: enhancing sustainability and reducing public health risks. Int. J. Adv. Multidiscip. Res. Stud. 4 (6), 1720–1729. 10.62225/2583049X.2024.4.6.4102

[B2] Alhomoud F. (2020). “Don’t let medicines go to waste”—A survey of pharmacists’ waste-reducing activities across GCC countries. Front. Pharmacol. 11, 1334. 10.3389/fphar.2020.01334 32982744 PMC7485414

[B3] Alqurshi A. (2020). Household disposal and recycling of medication in Saudi Arabia: a call for introducing medication take-back programs. Pharmacol. Pharm. 11 (11), 316–329. 10.4236/pp.2020.1111026

[B4] Aziz Z. Kim H. Al-Qadami A. S. Khan M. A. Ihsanullah I. Ahmad A. (2025). Pharmaceutical pollution in the aquatic environment: advanced oxidation processes as efficient treatment approaches. Mater. Adv. 6 (11), 3433–3454. 10.1039/D4MA01122H

[B5] Bano F. Prasad B. Kumari M. Meili L. Prasad K. S. (2025). Pharmaceuticals in water and wastewater: a review on ecological risk and sustainable treatment options. Water Air Soil Pollut. 236, 516. 10.1007/s11270-025-08181-x

[B6] Bekker C. L. Gardarsdottir H. Egberts A. C. G. Molenaar H. A. Bouvy M. L. van den Bemt B. J. F. (2019). What does it cost to redispense unused medications in the pharmacy? A micro-costing study. BMC Health Serv. Res. 19, 243. 10.1186/s12913-019-4065-6 31014325 PMC6481041

[B7] Blackburn J. Forrester C. Eii M. N. (2024). Reducing drug waste in hospitals. BMJ 386, e076200. 10.1136/bmj-2023-076200 39179292

[B8] IQVIA Institute for Human Data Science (2025). The global use of medicines 2025: outlook to 2029. Parsippany, NJ: IQVIA Institute.

[B9] Jafarzadeh A. Mahboub-Ahari A. Najafi M. Yousefi M. Dalal K. (2021). Medicine storage, wastage, and associated determinants among urban households: a systematic review and meta-analysis. BMC Public Health 21, 1127. 10.1186/s12913-021-06655-2 34118923 PMC8196539

[B10] Kardas P. Bennett B. Borah B. Burnier M. Daly C. Hiligsmann M. (2024). Medication non-adherence: reflecting on two decades since WHO adherence report and setting goals for the next twenty years. Front. Pharmacol. 15, 1444012. 10.3389/fphar.2024.1444012 39764461 PMC11700791

[B11] Khan M. Easwaran V. Orayj K. Venkatesan K. Shaik Alavudeen S. Alhadeer S. A. (2025). A cross-sectional study on perceptions toward safe disposal of unused/expired medicines in Saudi Arabia. PeerJ 13, e19258. 10.7717/peerj.19258 40292096 PMC12032961

[B12] Murray A. K. Hayes A. Snape J. Kasprzyk-Hordern B. Gaze W. H. Murray A. K. (2024). Co-selection for antibiotic resistance by environmental contaminants. Npj Antimicrob. Resist. 2, 9. 10.1038/s44259-024-00026-7 39843965 PMC11721650

[B13] Naseralallah L. Stewart D. Price M. Paudyal V. (2023). Prevalence, contributing factors, and interventions to reduce medication errors in outpatient and ambulatory settings: a systematic review. Int. J. Clin. Pharm. 45 (6), 1359–1377. 10.1007/s11096-023-01626-5 37682400 PMC10682158

[B14] National Center for Waste Management (NCWM) (2023). Technical guidelines—Healthcare waste management (TG WM 11). NCWM. Available online at: https://istitlaa.ncc.gov.sa.

[B15] OECD (2025). Pharmaceutical spending. OECD. Available online at: https://www.oecd.org/en/data/indicators/pharmaceutical-spending.html.

[B16] Saha K. Patel B. Paladini S. (2025). The role of leadership and cultural barriers in the adoption of lean six sigma in clinical pharmacy practice and medicine waste reduction. Int. J. Qual. Reliab. Manag. 42 (3), 809–836. 10.1108/IJQRM-02-2024-0069

[B17] Smale E. M. van den Bemt B. J. F. Heerdink E. R. Desar I. M. E. Egberts T. C. G. Bekker C. L. (2024). Cost savings and waste reduction through re-dispensing unused oral anticancer medications: the ROAD study. JAMA Oncol. 10 (1), 87–94. 10.1001/jamaoncol.2023.4865 37971730 PMC10654927

[B18] Swan G. Naidoo V. Cuthbert R. Green R. E. Pain D. J. Swarup D. (2006). Removing the threat of diclofenac to critically endangered Asian vultures. PLoS Biol. 4 (3), e66. 10.1371/journal.pbio.0040066 16435886 PMC1351921

[B19] U.S. Environmental Protection Agency (EPA) (2025). The impact of pharmaceuticals released to the environment. U. S. EPA. Available online at: https://www.epa.gov/household-medication-disposal/impact-pharmaceuticals-released-environment.

[B20] Wang L. S. Aziz Z. Wang E. S. Chik Z. (2024). Unused medicine take-back programmes: a systematic review. J. Pharm. Policy Pract. 17 (1), 2395535. 10.1080/20523211.2024.2395535 39257836 PMC11385643

[B21] Wang L. S. Aziz Z. Wang H. J. Chik Z. (2025). Prevalence and disposal of unused medicines: a systematic review of cross-sectional studies. J. Pharm. Health Serv. Res. 16 (1), rmae028. 10.1093/jphsr/rmae028

[B22] Watts S. Coutsouvelis J. Wickens J. Poole S. Percival M. Zalcberg J. R. (2024). Medication reuse programs: a narrative review of the literature. Int. J. Qual. Health Care 36 (2), mzae036. 10.1093/intqhc/mzae036 38687831

[B23] World Health Organization (WHO) (2023). Anatomical therapeutic chemical (ATC) classification: toolkit and guidelines. Available online at: https://www.who.int/tools/atc-ddd-toolkit/atc-classification (Accessed January 9, 2026).

[B24] World Health Organization (WHO) (2025). Safe management of pharmaceutical waste from health care facilities: global best practices.

